# Immunization with a dominant-negative recombinant Herpes Simplex Virus (HSV) type 1 protects against HSV-2 genital disease in guinea pigs

**DOI:** 10.1186/1471-2180-10-163

**Published:** 2010-06-03

**Authors:** Richard Brans, Feng Yao

**Affiliations:** 1 Department of Surgery, Brigham and Women's Hospital, and Harvard Medical School, Boston, MA 02115, USA; 2 Department of Dermatology and Allergology, University Hospital of the RWTH Aachen, 52074 Aachen, Germany

## Abstract

**Background:**

CJ9-gD is a novel dominant-negative recombinant herpes simplex virus type 1 (HSV-1) that is completely replication-defective, cannot establish detectable latent infection *in *vivo, and expresses high levels of the major HSV-1 antigen glycoprotein D immediately following infection. In the present study, CJ9-gD was evaluated as a vaccine against HSV-2 genital infection in guinea pigs.

**Results:**

Animals immunized with CJ9-gD developed at least 700-fold higher titers of HSV-2-specific neutralization antibodies than mock-immunized controls. After challenge with wild-type HSV-2, all 10 control guinea pigs developed multiple genital lesions with an average of 21 lesions per animal. In contrast, only 2 minor lesions were found in 2 of 8 CJ9-gD-immunized animals, representing a 40-fold reduction on the incidence of primary genital lesions in immunized animals (p < 0.0001). Immunization significantly reduced the amount and duration of viral shedding and provided complete protection against neurological symptoms, while 90% of mock-immunized animals succumbed due to the severity of disease. Importantly, immunized animals showed no signs of recurrent disease or viral shedding during a 60-days observation period after recovery from primary infection, and carried 50-fold less latent viral DNA load in their dorsal root ganglia than the surviving mock-vaccinated controls (p < 0.0001).

**Conclusions:**

Collectively, we demonstrate that vaccination with the HSV-1 recombinant CJ9-gD elicits strong and protective immune responses against primary and recurrent HSV-2 genital disease and significantly reduces the extent of latent infection.

## Background

Genital herpes is the main cause of genital ulcer disease worldwide and is due to infections with herpes simplex virus (HSV) [1,2]. HSV-2 accounts for most cases of genital herpes [3]. Recent studies indicate that in developed countries HSV-1 has become the main causative agent for primary genital herpes, especially among adolescents, women, and homosexual men [4-7]. The prevalence of HSV-2 in the general population ranges from 10%-60%, indicating that genital herpes is one of the most common sexually transmitted diseases [2,8].

After primary genital infection, HSV establishes latent infection in dorsal root ganglia with lifelong persistence, subsequently giving rise to intermittent reactivation and recurrent disease [9]. As the clinical appearance of genital HSV infection varies from unspecific symptoms to typically painful lesions [10], only 10-25% of people who are seropositive for HSV-2 are aware that they have genital herpes. HSV is intermittently shed from the genital mucosa in the absence of symptoms causing subconscious transmission of disease [11]. Vertical transmission of HSV to neonates is associated with a high mortality rate and a high incidence of neurological sequelae in survivors [12]. In addition, genital herpes has been linked to an increased risk of sexually acquiring and transmitting human immunodeficiency virus (HIV), which can be strongly reduced by HSV antiviral therapy [13,14]. To date, the treatment and prevention of primary and recurrent disease is limited [15]. Experimental vaccine approaches against genital herpes have included peptides, proteins, killed virus, DNA vaccines, heterologous replicating viral vectors, replication-defective viruses, and attenuated replication-competent viruses [16,17]. Considering the general impact of HSV-1 diseases and rising importance of primary genital herpes caused by HSV-1, a desirable vaccine should be capable of offering effective protective immunity against both HSV subtypes.

A main target for subunit vaccine development has been HSV glycoprotein D (gD), a major antigen on the viral envelope [17]. Subunit vaccines containing gD in combination with an adjuvant appeared to be safe and effective against genital herpes in guinea pigs [18-20], but failed to provide general protection in clinical trials [21,22]. Replication-defective viruses lacking functions essential for viral replication or assembly of progeny virus particles have a broad antigenic spectrum and are more efficient than subunit vaccines in eliciting protective immune responses against genital HSV in mice and guinea pigs [23]. However, the use of replication-defective viruses, particularly when used in latently infected individuals, imposes certain risks, as they might regain replication competence in the presence of wild-type virus or reactivate latent wild-type virus infections [24].

To minimize these safety concerns, using the T-REx™ gene switch technology (Invitrogen, Carlsbad, CA) developed in our laboratory and the dominant-negative mutant polypeptide UL9-C535C of HSV-1 origin binding protein UL9, we generated a novel class of replication-defective HSV-1 recombinant, CJ83193, which can prevent its own viral DNA replication as well as that of wild-type HSV-1 and HSV-2 in co-infected cells [25,26]. To increase its safety and vaccine efficacy against HSV infections, we recently constructed a CJ83193-derived HSV-1 recombinant CJ9-gD by replacing the essential UL9 gene with an extra copy of the HSV-1 gD (gD1) gene under the control of the tetO-bearing hCMV major immediate-early promoter [27]. We demonstrated that unlike the gD gene controlled by the endogenous promoter whose expression is dependent on viral replication [28], CJ9-gD expresses high-levels of gD at the immediate-early phase of HSV infection. Moreover, CJ9-gD is completely replication-defective and causes no detectable infection in trigeminal ganglia after ocular or nasal infection in mice [27]. In mice, CJ9-gD induces strong and long-lasting humoral and Th1-associated cellular immune responses against HSV-1 and HSV-2 [27,29]. Immunization with CJ9-gD protects mice against HSV-1 ocular keratitis and guinea pigs against HSV-1 skin disease [27,30] as well as genital herpetic disease caused by wild-type HSV-1 and HSV-2 in mice [29]. Previously, we have shown further that CJ9-gD is a safer and more effective vaccine than non-gD-expressing parental CJ83193 virus against HSV-1 infection [27,29].

The guinea pig model of HSV-2 genital infection offers a unique advantage over the mouse model to investigate the efficacy of candidate HSV vaccine in protection against primary and recurrent HSV-2 genital infection and disease. Specifically, following primary intravaginal infection with HSV-2, guinea pigs develop vesicular lesions resembling those in humans, including development, appearance, and duration of disease. In contrast to mice in which spontaneous reactivation from latent infection rarely occurs in the vaginal tract, guinea pigs undergo episodic spontaneous recurrent infection and disease after recovering from initial genital disease [31,32]. In the current report, we investigate whether CJ9-gD can serve as an effective vaccine in protection against both primary and recurrent HSV-2 genital infection and disease in guinea pigs following intravaginal challenge with wild-type HSV-2.

## Results

### Induction of HSV-2-specific neutralization antibodies

**Figure 1:**
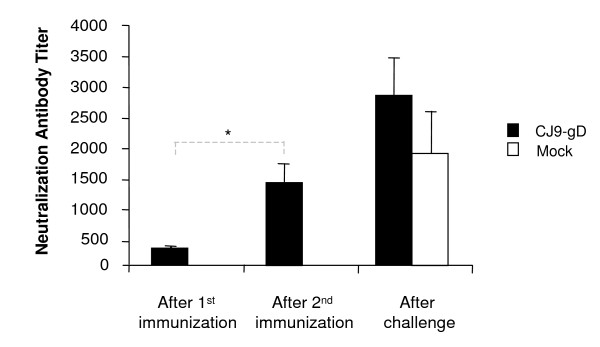
**Induction of HSV-2-specific neutralizing antibodies in immunized guinea pigs**. Two sets of guinea pigs (n = 8; n = 10) were injected s.c. with 5 × 10^6 ^PFU/animal of CJ9-gD or with DMEM and boosted after 3 weeks. Blood was taken 3 weeks after each immunization and 5 weeks after challenge. After heat inactivation, serum from each animal was assayed separately for HSV-2-specific neutralizing antibody titers on Vero cell monolayers. The results represent average titers ± SEM. P-value was assessed by Student's t-test (* p < 0.005).

The ability of CJ9-gD to elicit HSV-2-specific neutralizing antibodies was determined (Fig. 1). The HSV-2-specific neutralization antibody titer was detected in serum from all immunized guinea pigs and increased significantly from the first to the second vaccination (p < 0.005) with a peak titer 3 weeks after the second vaccination of 1400. No HSV-2-specific neutralization antibody was detected in serum from mock-immunized animals at 1:2-dilution before challenge. After challenge with the wild-type HSV-2, the neutralization antibody titer in immunized animals increased 2-fold (p > 0.05) and was 1.5-fold higher than that in mock-immunized controls following challenge.

### Reduction of intravaginal replication of challenge HSV-2 in immunized guinea pigs

**Figure 2:**
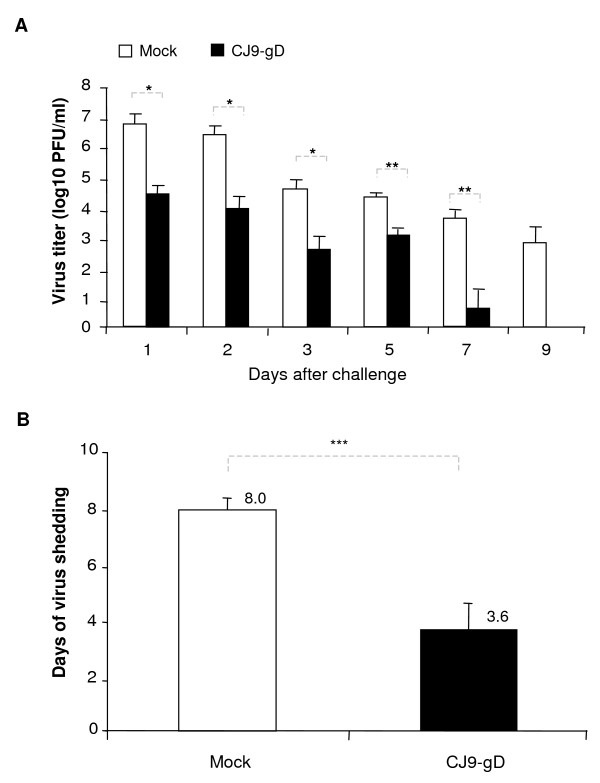
**Reduction of challenge HSV-2 vaginal replication in guinea pigs immunized with CJ9-gD**. One set of 8 and one set of 10 guinea pigs were inoculated s.c. with either 5 × 10^6 ^PFU/animal of CJ9-gD or DMEM and boosted after 3 weeks. At 6 weeks guinea pigs were challenged intravaginally with 5 × 10^5 ^PFU of HSV-2 strain MS. Vaginal swabs were taken on days 1, 2, 3, 5, 7, and 9 post-challenge. Infectious virus in swab materials was assessed by standard plaque assay on Vero cell monolayers. Viral titers are expressed as the mean ± SEM in individual vaginal swabs (A). The duration of viral shedding is represented as the mean number of days during which infectious virus was detected in swab materials following challenge ± SEM (B). P-values were assessed by Student's t-test (* p < 0.05, ** p < 0.005, *** p < 0.0005)

Six weeks after the initial immunization, two sets of guinea pigs (n = 8; n = 10), which were either mock-immunized (n = 10) or immunized with 5 × 10^6 ^PFU of CJ9-gD (n = 8), were challenged intravaginally with wild-type HSV-2 strain MS. To examine the effectiveness of immunization with CJ9-gD against intravaginal replication of challenge HSV-2, vaginal swabs were taken on days 1, 2, 3, 5, 7 and 9 after challenge. As shown in Fig. 2A, the yields of challenge virus were significantly lower in immunized guinea pigs compared with those in mock-immunized controls from days 1 to 7 (p-values for days 1, 2, and 3 < 0.05, p-value for days 5 and 7 < 0.005), with a reduction of 207-fold on day 1 (p = 0.036) and 220-fold on day 2 (p = 0.012). By day 9 no challenge virus was detected in CJ9-gD-immunized guinea pigs, whereas 50% of mock-immunized animals continued to shed virus at an overall average yield of more than 7.1 × 10^2 ^PFU/ml. Compared with mock-immunized controls, the average duration of viral shedding in immunized guinea pigs decreased markedly from more than 8 days to 3.6 days (Fig. 2B, p < 0.0005).

### Protection against primary HSV-2 genital disease in immunized guinea pigs

After intravaginal challenge with wild-type HSV-2, animals were monitored daily for signs of disease. The development and clinical appearance of lesions caused by challenge virus in mock-vaccinated guinea pigs was consistent with previous observations.

**Figure 3:**
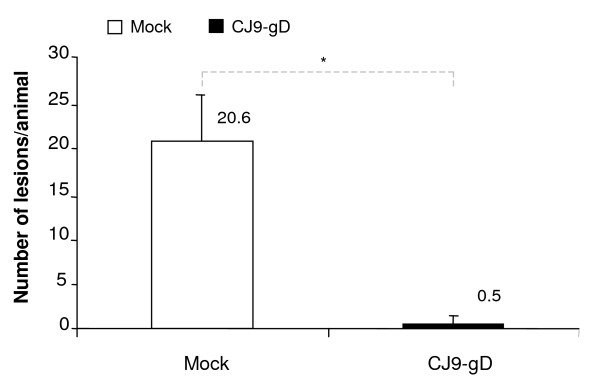
**Prevention of primary HSV-2 genital lesions in guinea pigs immunized with CJ9-gD**. Mock-immunized and CJ9-gD-immunized guinea pigs described in Fig. 2 were monitored daily for clinical symptoms following challenge with wild-type HSV-2. The average number of lesions per immunized animals was compared with that found in mock-immunized guinea pigs. The indicated values represent the mean number of lesions ± SD on day 6 post-challenge. P-value was assessed by Student's t-test (* p < 0.0001).

**Figure 4:**
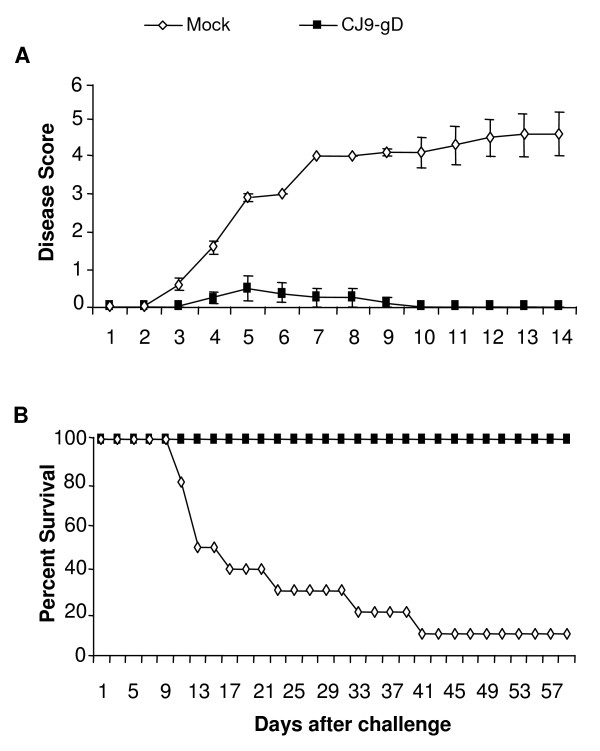
**Prevention of primary HSV-2 disease in guinea pigs immunized with CJ9-gD**. After challenge with wild-type HSV-2, individual guinea pigs described in legend of Fig. 3 were observed during a 60-day follow-up period for the incidence of genital and disseminated HSV-2 disease using the following score: 0 = no disease; 1 = redness or swelling; 2 = a few small vesicles; 3 = several large vesicles; 4 = several large ulcers with maceration; 5 = paralysis; and 6 = death. Presented is the disease score for the first 15 days after challenge (A.) and the percentage of survival until day 60 after challenge (B.).

The impact of immunization with CJ9-gD on the incidence of skin lesions is summarized in Fig. 3. All 10 mock-immunized guinea pigs (100%) developed multiple genital herpes lesions following challenge with wild-type HSV-2. In contrast, only 2 of 8 animals immunized with 5 × 10^6 ^PFU of CJ9-gD exhibited two mild herpetiform lesions, resulting in an average of 0.5 lesions per immunized animal. In the corresponding control group, an average of 20.6 lesions per mock-vaccinated animal was detected on day 6 post-challenge (p < 0.0001). Thus, the overall incidence of primary herpetic skin lesions in immunized animals was reduced 40-fold compared to mock-immunized controls. Moreover, the two lesions found in the above mentioned immunized guinea pigs were less pronounced and healed off within 2 to 6 days, whereas in mock-immunized animals lesions turned into macerating ulcers with prolonged healing (Fig. 4A) and associated with systemic spreading of virus. All immunized guinea pigs survived the study and showed no signs of neurological illness, whereas 5 of 10 mock-immunized animals (50%) were sacrificed by day 14 after challenge due to hind limb paralysis and severity of disease. The mortality rate in this group increased to 90% by day 41 after challenge (Fig. 4B).

### Protection against recurrent HSV-2 infection in immunized guinea pigs

After recovery from intravaginal challenge with wild-type HSV-2, surviving animals were monitored daily from day 30 to day 60 for signs of recurrent disease. In addition, vaginal swabs were taken daily and assayed for the presence of infectious virus. All immunized animals, and 3 of the 10 mock-immunized controls that survived the first 30 days following challenge with wild-type HSV-2 were monitored for recurrent HSV-2 infection. Two of the mock-immunized animals had recurrent viral shedding between days 30 and 60, whereas one had recurrent lesions. In contrast, no lesions or recurrent viral shedding were detected in immunized guinea pigs (Table 1).

**Table 1:** Prevention of recurrent HSV-2 infection in guinea pigs immunized with CJ9-gD

	Mock (n = 3)	CJ9-gD (n = 8)
Recurrency^1^	3/3	0/8
Recurrent lesions^2^	1/3	0/8
Recurrent shedding^3^	2/3	0/8

^1 ^Overall number of guinea pigs with recurrent lesions and/or recurrent shedding between days 30 and 60 after challenge.

^2 ^Number of guinea pigs with recurrent genital lesion between days 30 and 60 after challenge.

^3 ^Number of guinea pigs from which virus was detected in vaginal swab material by plaque assay on Vero cell monolayers between days 30 and 60 after challenge.

### Protection from latent viral infection in immunized guinea pigs

**Figure 5:**
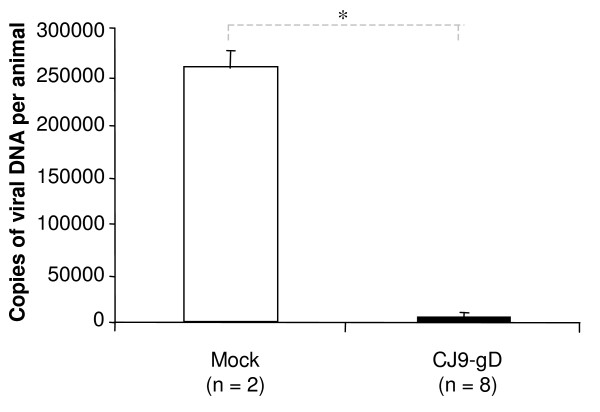
**Protection from latent viral infection in guinea pigs immunized with CJ9-gD**. Sixty days after challenge, 12 lower lumbar and sacral dorsal root ganglia (DRG) per guinea pig were harvested from all 8 immunized guinea pigs and the 2 surviving mock-immunized controls. The whole DNA was extracted and quantified for the presence of latent viral DNA using quantitative real-time PCR. The amount of viral DNA per guinea pig (A) was determined. The results are indicated as mean values ± SEM. P-value was assessed by Student's t-test (* p < 0.0001).

At day 60 after challenge, 12 lower lumbar and sacral dorsal root ganglia per guinea pig were harvested from all 8 animals immunized with CJ9-gD and the 2 surviving mock-immunized controls. The whole DNA was extracted and tested for latent viral DNA using quantitative real-time PCR. The limit of detection was 5 DNA copies per reaction (correlation coefficient +/- SD: 0.96 +/- 0.016). As shown in Fig. 5, the average amount of latent HSV DNA per guinea pig was 50-fold greater in mock-vaccinated controls than in immunized animals (261486 DNA copies vs. 5229 DNA copies, p ≤ 0.0001).

## Discussion

Although to date no vaccine capable of completely preventing HSV infection has been reported, it is believed that great benefits can be obtained from developing a vaccine that prevents disease with or without partial protection from infection as demonstrated with pertussis and influenza virus vaccines [34]. Our earlier studies demonstrate that immunization with CJ9-gD induces strong and long-lasting HSV-1- as well as HSV-2-specific humoral and Th1- cellular immune responses in mice, leading to a significant reduction in the amount and duration of acute replication of wild-type HSV-1 and HSV-2 after vaginal challenge compared with mock-immunized controls. At an immunization dose of 2 × 10^6 ^PFU of CJ9-gD, mice were completely protected from HSV-1 and HSV-2 disease [29]. We were, however, unable to evaluate whether immunization with CJ9-gD is effective in protection against recurrent HSV genital infection and disease in mice. Therefore, in the present report we used guinea pigs to explore the efficacy of immunization with CJ9-gD against HSV-2 primary as well as recurrent genital infection and disease.

We demonstrate that immunization with CJ9-gD at a dose of 5 × 10^6 ^PFU elicits high levels of neutralizing antibodies against HSV-2 in guinea pigs. Titers increased significantly from the first to the second vaccination, indicating a boosting effect. Like in mice [29], immunization with CJ9-gD induced about 7-fold higher neutralization antibody titers in guinea pigs against HSV-1 than HSV-2 (p < 0.0001) (data not shown). In the present study cellular immune responses were not tested due to the lack of sufficient immunological reagents specific for guinea pigs. We did, however, demonstrate in mice that immunization with CJ9-gD elicits strong HSV-specific CD4^+ ^and CD8^+ ^T-cell responses against HSV-1 and in a lesser extent against HSV-2 at levels similar or comparable to those induced by wild-type HSV-1 [27,29]. Given the demonstrated importance for both HSV-specific CD4^+ ^and CD8^+ ^T cells in clearance of virus from infected epithelium and in neural tissues in the mouse model of HSV infections [35-40], it is not surprising that HSV-2 cross-specific-T cell response elicited in CJ9-gD-immunized guinea pigs plays a critical role in limiting the replication of challenge virus during both primary infection and reactivation from latency.

Immunization with CJ9-gD significantly reduced the amount and duration of wild-type virus replication as well as the number of genital lesions after vaginal challenge with HSV-2 compared with that in mock-immunized guinea pigs. Only 2 of 8 immunized animals developed 2 mild and fast healing herpetiform lesions with no signs of systemic involvement. Morbidity was quite extensive in mock-vaccinated animals with an average of 20.6 lesions per animal, a high incidence of systemic involvement, and a mortality rate of 90%. High mortality rates in mock-vaccinated animals after vaginal challenge with wild-type HSV-2 have been reported by other groups [19,41] and limit the evaluation of recurrences. The extent of disease might be influenced by the viral strain or stock, the viral titer and by the inoculation method used. Despite the extensive disease in mock-vaccinated animals, CJ9-gD provided good protection against genital challenge with wild-type HSV-2 in immunized guinea pigs. Therefore, it is reasonable to anticipate that protection would be more effective should a lower dose of challenge virus or a more gentle inoculation be selected.

In accordance with the protection against primary disease, neither recurrent vaginal shedding of infectious virus nor recurrent genital lesions were found in CJ9-gD-immunized animals. Quantitative PCR analysis shows that the amount of latent HSV DNA in dorsal root ganglia was 50-fold lower in immunized guinea pigs compared with the 2 mock-immunized guinea pigs that survived following challenge with wild-type HSV-2 (p < 0.0001). Recall that CJ9-gD cannot establish detectable latent infection in sensory ganglia in mice following ocular or intranasal infection [27] nor in dorsal root ganglia after subcutaneous immunization [29]. The viral DNA detected in dorsal root ganglia of CJ9-gD-immunized guinea pigs after vaginal challenge should be primarily the challenge wild-type HSV-2 viral DNA. Taken together, these results are consistent with observations that a reduced latent infection is associated with a lower incidence of reactivation and recurrent disease [20,41,42].

Several vaccine candidates have been tested in guinea pigs against genital HSV-2 infection. The subunit vaccine gD2/AS04, which contains the HSV-2 major antigen glycoprotein D (gD2) in combination with the adjuvant aluminium hydroxide and 3-O-deacylated-monophosphoryl lipid A (MPL), was effective in prevention of primary and recurrent genital disease in immunized animals following challenge with wild-type HSV-2 [19,20]. In phase 3 clinical trials, however, it only provided 73-74% efficacy in protecting against development of genital herpes disease in HSV-seronegative women [21]. A recent study showed that the replication-defective HSV-2 recombinant *dl*5-29 was more effective than the HSV-2-gD-based subunit vaccine in inducing HSV-2-specific neutralizing antibodies and CD8^+ ^T-cell response in mice [43]. CJ9-gD is an HSV-1 recombinant defective at level of viral DNA replication, and therefore, similar to dl5-29, capable of expressing a broad spectrum of viral antigens. In addition, it has a unique dominant-negative effect on viral replication (UL9-C535C expression) and expresses high levels of the major HSV-1 antigen gD at the immediate-early phase of infection [27]. Immunization with CJ9-gD led to 220-fold reduction in the yield of challenge wild-type HSV-2 in genital swabs materials on day 2 post-challenge compared with mock-immunized controls. Noting that immunization with gD2/AS04 resulted in less than 14-fold challenge wild-type HSV-2 (strain MS) viral replication compared with mock-immunized controls on day 2 post-challenge, and all mock-immunized animals survived after recovery from primary disease caused by challenge virus [20], our study suggests that CJ9-gD could potentially be more efficacious than gD2 subunit vaccine against HSV-2 genital disease. It will be interesting to test the vaccine efficacy of gD2/AS04 and CJ9-gD in protecting against HSV-2 genital herpes in the same experimental settings. Moreover, in light of that CJ9-gD expresses high-level of gD, and induction of both effective mucosal and systemic immune responses is likely required for an optimal protection against HSV genital infection, it would be of great interest to investigate the effectiveness of CJ9-gD in induction of humoral and T-cell immunity following different routes of immunization and whether the efficacy of CJ9-gD in eliciting mucosal immune response can be enhanced by gD subunit prime/CJ9-gD boost regimen involving combination of mucosal and systemic immunization [44-46].

Many type-common and type-specific antibodies as well as T cell epitopes have been identified against various HSV-1 and HSV-2 proteins. Mice immunized with CJ9-gD develop stronger humoral and cellular immune responses against HSV-1 than against HSV-2, and are significantly better protected against genital infection with HSV-1 than with HSV-2 [29]. These findings are in agreement with the previous reports that in rodents HSV vaccines are generally less effective in prevention of heterotypic HSV infection than homotypic infection [47,48]. Combined with observations that humans who were previously infected with HSV-2 are less likely to experience re-infection with a heterologous strain of HSV-2 than individuals with prior HSV-1 infection [49-53], it is reasonable to believe, that a CJ9-gD-like dominant-negative HSV-2 recombinant would be more effective in prevention of genital HSV-2 infection than the HSV-1 recombinant CJ9-gD. This HSV-2 recombinant is currently being devised.

## Conclusions

We demonstrate that immunization with a replication-defective and dominant-negative HSV-1 recombinant CJ9-gD expressing high levels of gD can induce strong cross-protective immunity against primary and recurrent HSV-2 genital infection and disease in guinea pigs. We show further that the latent viral load of challenge wild-type HSV-2 is significantly reduced in immunized guinea pigs compared with the mock-immunized controls. Collectively, CJ9-gD represents a new class of HSV-1 recombinant, which is avirulent, unable to establish detectable latent infection *in vivo*, and serves as an effective vaccine against genital HSV infection and disease in both mice and guinea pigs.

## Methods

### Animals

Female Hartley guinea pigs (300-350 g) were obtained from Charles River Breeding Laboratories (Wilmington, MA). The described animal experiments were conducted according to the protocols approved by the Harvard Medical Area Standing Committee on Animals and the American Veterinary Medical Association. The Harvard Medical School animal management program is accredited by the Association for Assessment and Accreditation of Laboratory Animal Care (AAALAC) and meets National Institutes of Health standards as set forth in "The Guide for the Care and Use of Laboratory Animals" (National Academy Press, 1996).

### Cells and viruses

African Green Monkey Kidney (Vero) cells and RUL9-8 cells, a cell line derived from U2OS cells expressing UL9 and the tetracycline repressor (tetR), were grown and maintained in DMEM growth medium as previously described [33].

Wild-type HSV-2 MS strain (ATCC, Manassas, VA) was propagated and plaque assayed on Vero cells. CJ9-gD was derived from CJ83193 by replacing the essential UL9 gene with the HSV-1 gD gene driven by the tetO-containing hCMV major immediate-early promoter [27]. CJ83193 is a replication-defective virus, in which both copies of the HSV-1 ICP0 gene were replaced by DNA sequences encoding the dominant-negative HSV-1 polypeptide UL9-C535C under control of the tetO-bearing hCMV major immediate-early promoter [25]. CJ9-gD was propagated and plaque assayed in RUL9-8 cells.

### Immunization and challenge

One set of 8 guinea pigs and one set of 10 guinea pigs were randomly assigned to 2 groups. Animals were either mock-immunized with DMEM (n = 10) or immunized with 5 × 10^6 ^PFU of CJ9-gD (n = 8) in a volume of 50 μl s.c. in the right and left upper flank per guinea pig. On day 21 after primary immunization, animals were boosted. At the same time and one day prior to viral challenge, serum was obtained from saphenous veins and stored at -80°C. Six weeks after the initial immunization, the animals were preswabbed with a moist sterile calcium alginate swab (Fisher Scientific, Waltham, MA) and inoculated intravaginally with 100 μl of culture medium containing 5 × 10^5 ^PFU of HSV-2 strain MS. Animals were kept on their backs with their rear part elevated under the influence of anesthesia for 30-45 min post-infection.

### Detection of HSV-2-specific neutralization antibody titers

Blood was obtained from the saphenous veins and neutralization antibody titers were determined in the presence of complement as described previously [28,30].

### Clinical observations

After challenge with wild-type HSV-2 strain MS, the animals were monitored daily until day 60. The number of lesions were counted and the progress of disease was scored using a modified method [31]: 0 = no disease; 1 = redness or swelling; 2 = a few small vesicles; 3 = several large vesicles; 4 = several large ulcers with maceration; 5 = paralysis; and 6 = death.

### Assay of acute and recurrent vaginal shedding of challenge virus

After challenge with wild-type HSV-2 strain MS, vaginal mucosae were swabbed with a moist calcium alginate swab (Fisher Scientific, Waltham, MA) on days 1, 2, 3, 5, 7 and 9. From days 30 to 60 post challenge swabs were taken daily. Swabs were kept in 1 ml DMEM and stored at -80°C until assayed for infectious virus by standard plaque assay on Vero cell monolayers.

### Quantitative real-time PCR

At day 60 after intravaginal challenge with HSV-2 strain MS, 12 lower lumbar and sacral dorsal root ganglia were collected from each of the surviving guinea pigs. Dorsal root ganglia were kept separately in 0.5 ml of normal growth medium and stored at -80°C for further processing. DNA was isolated from each dorsal root ganglion and assayed for viral DNA by quantitative real-time PCR as described previously [27].

### Statistical analysis

For statistical analysis unpaired Student's t-tests were performed.

## Abbreviations

HSV: Herpes simplex virus; gD: Glycoprotein D.

## Competing interests

The authors declare that they have no competing interests.

## Authors' contributions

RB participated in designing the experiments, carried out the animal studies, cell culture work, virus assays, and drafted the manuscript. FY developed the HSV-1 recombinant CJ9-gD, designed the experiments, and participated in their coordination and drafting the manuscript. Both authors read and approved the final manuscript.
